# The CCAAT box-binding transcription factor NF-YA1 controls rhizobial infection

**DOI:** 10.1093/jxb/ert392

**Published:** 2013-12-06

**Authors:** Philippe Laporte, Agnes Lepage, Joëlle Fournier, Olivier Catrice, Sandra Moreau, Marie-Françoise Jardinaud, Jeong-Hwan Mun, Estibaliz Larrainzar, Douglas R. Cook, Pascal Gamas, Andreas Niebel

**Affiliations:** ^1^INRA, Laboratoire des Interactions Plantes-Microorganismes (LIPM), UMR441, Castanet-Tolosan, F-31326, France; ^2^CNRS, Laboratoire des Interactions Plantes-Microorganismes (LIPM), UMR2594, Castanet-Tolosan, F-31326, France; ^3^INPT-Université de TOULOUSE, ENSAT-Avenue de l’Agrobiopole, Auzeville-Tolosane, 31326-Castanet-Tolosan Cedex, France; ^4^Department of Agricultural Biotechnology, National Academy of Agricultural Science, Rural Development Administration, 150 Suin-ro, Gwonseon-gu, Suwon 441-707, Korea; ^5^Department of Bioscience and Bioinformatics, College of Natural Science, Myongji University, Seoul, Korea; ^6^Department of Plant Pathology, University of California, Davis, CA 95616, USA; * Present adresss: Dpto. Ciencias del Medio Natural, Universidad Pública de Navarra, CampusArrosadia 31006 Pamplona, Spain.

**Keywords:** CCAAT box-binding factor, infection, infection thread, *Medicago truncatula*, NF-YA, nodule development, rhizobium, symbiosis.

## Abstract

Symbiosis between legume plants and soil rhizobia culminates in the formation of a novel root organ, the ‘nodule’, containing bacteria differentiated as facultative nitrogen-fixing organelles. MtNF-YA1 is a *Medicago truncatula* CCAAT box-binding transcription factor (TF), formerly called HAP2-1, highly expressed in mature nodules and required for nodule meristem function and persistence. Here a role for MtNF-YA1 during early nodule development is demonstrated. Detailed expression analysis based on RNA sequencing, quantitiative real-time PCR (qRT-PCR), as well as promoter–β-glucuronidase (GUS) fusions reveal that *MtNF-YA1* is first induced at the onset of symbiotic development during preparation for, and initiation and progression of, symbiotic infection. Moreover, using a new knock-out mutant, *Mtnf-ya1-1*, it is shown that *MtNF-YA1* controls infection thread (IT) progression from initial root infection through colonization of nodule tissues. Extensive confocal and electronic microscopic observations suggest that the bulbous and erratic IT growth phenotypes observed in *Mtnf-ya1-1* could be a consequence of the fact that walls of ITs in this mutant are thinner and less coherent than in the wild type. It is proposed that *MtNF-YA1* controls rhizobial infection progression by regulating the formation and the wall of ITs.

## Introduction

Plants within the legume family possess the remarkable pro- perty of endosymbiotic interaction with a group of bacteria collectively referred to as ‘rhizobia’. Symbiotic development culminates in the formation of a new root organ called the nodule, which is colonized by rhizobia that fix atmospheric dinitrogen to ammonia, which is then assimilated by the host plant. A molecular dialogue between the two partners initiates nodule development. Beginning prior to infection, a phase often referred to as the pre-infection phase, plant-derived flavonoids stimulate rhizobia to secrete lipochito-oligosaccharide signal molecules called Nod factors (NFs), which are essential for the initiation of symbiotic infection and nodule organogenesis. NFs reorient root hair growth to induce a curl or ‘shepherd’s crook’. Entrapped within the curl, or sometimes between two adjacent root hairs, a microcolony of rhizobia is formed (Gage, 2004) from which rhizobia enter the root hair following local degradation of the root hair cell wall (Ridge and Rolfe, 1985; van Spronsen *et al.*, 1994), a process that involves both rhizobial cellulase and legume pectate lyase genes (Robledo *et al.*, 2008; Xie *et al.*, 2012). Bacterial entry is accompanied by remodelling of the plasma membrane and primary root hair cell wall (Brewin, 2004; Robledo *et al.*, 2008), leading to the formation of a tube-like apoplastic compartment called the infection thread (IT). Bacteria migrate through the IT’s extracellular matrix towards the root cortex and developing nodule organ by a combination of cell divisions and sliding movements (Fournier *et al.*, 2008). The IT extracellular matrix contains plant glycoproteins, as is typical of extracellular matrices, while the IT wall is similar in composition to that of other plant cell walls, containing cellulose, xyloglucans, and methyl-esterified and non-esterified pectins (Rae *et al.*, 1992). Coincident with infection, cell divisions are activated in root cortical cells that subtend the growing IT. Early cell divisions signify the nascent nodule primordium that subsequently develops into a fully active meristematic tissue. In legumes such as *Medicago truncatula*, cell divisions are initially observed in pericycle cells (Timmers *et al.*, 1999) but then predominantly in inner cortical cells, and the resulting indeterminate meristem drives nodule growth. In their mature state, indeterminate nodules are composed of characteristic zones of development, with each zone composed of specialized tissues and cell types (Vasse *et al.*, 1990). Zone 1 is the apical zone, characterized by meristematic activity and the absence of rhizobia. Zone 2 is the pre-fixation zone containing numerous ITs that continually re-infect meristem-derived cells and from which bacteria are released into small cytoplasmic vesicles known as symbiosomes. Interzone 2–3 is a narrow, amyloplast-rich cell layer preceding full nitrogen fixation and within which key plant and bacterial symbiotic genes are activated (Soupene *et al.*, 1995). Zone 3 is the fixation zone or nodule ‘central tissue’, where host cells and rhizobia complete differentiation processes that were initiated in the proximal part of zone 2, including endoreduplication and the acquisition of morphological features characteristic of the nitrogen-fixing organelle, the ‘bacteroid’.

Forward and reverse genetic approaches have identified genes and contribute to knowledge of the signalling pathway(s) required for successful infection and nodule organogenesis (Murray, 2011; Oldroyd, 2013). The perception of NF by LysM receptor kinases, including Nod factor perception (MtNFP) (Ben Amor *et al.*, 2003; Arrighi *et al.*, 2006), and the subsequent generation and deciphering of nuclear calcium spikes, lead to a developmental cascade that also involves new host gene transcription. Signalling and symbiotic development downstream of NF receptors requires the anion channel *Doesn’t make infection1* (DMI1) (Ane *et al.*, 2004), the receptor kinase DMI2 (Catoira *et al.*, 2000; Endre *et al.*, 2002), and the calcium calmodulin kinase DMI3 (Mitra *et al.*, 2004a). A second LysM receptor kinase, LYK3, is essential for proper root hair curling and IT progression (Catoira *et al.*, 2001; Smit *et al.*, 2007). Several genes encoding transcription factors (TFs) are regulators of rhizobial infection and progression, including *Nodulation signaling pathway1* (*NSP1*) and *NSP2*, two GRAS TFs (Oldroyd and Long, 2003; Smit *et al.*, 2005), *ERF required for Nodulation* 1 (*ERN1*) and *ERN2*, and *nodule inception* (*NIN*). Mutants in these genes are typically blocked at the microcolony stage and have absent or significantly reduced numbers of epidermal ITs. The nuclear protein IPD3 (called Cyclops in *Lotus japonicus*), a phosphorylation substrate of DMI3, is also essential for rhizobial colonization (Yano *et al.*, 2008; Horvath *et al.*, 2011) as is the case for the nuclear coiled-coil protein RPG (Arrighi *et al.*, 2008) and the Ubox/WD40 protein LUMPY INFECTIONS (LIN) (Kiss *et al.*, 2009).

Here the involvement of another type of TF during rhizobial infection, the CCAAT box-binding factor MtNF-YA1, is shown. MtNF-YA1 belongs to a family of TFs called nuclear factor Y (NF-Y), also called CCAAT box-binding factors (CBFs) or haem adhesion proteins (HAPs). NF-Y homologues occur in all eukaryotes. NF-Y binds CCAAT boxes in promoters as a heterotrimeric complex, composed of a specific CCAAT box-binding factor known as NF-YA and two histone-like proteins NF-YB and NF-YC. While in animals each subunit is encoded by a single gene, in plants structural diversification has led to the appearance of gene families of ~10 members, each with specialized functions in diverse developmental or stress-responsive processes (for reviews, see Petroni *et al.*, 2012; Laloum *et al.*, 2013). In previous reports, MtNF-YA1 (formerly called MtHAP2-1; see Laloum *et al.*, 2013) was identified as a rhizobial-induced, symbiosis-specific NF-YA subunit. *MtNF-YA1* is expressed in zones 1 and 2 of mature root nodules (El Yahyaoui *et al.*, 2004; Combier *et al.*, 2006; Moreau *et al.*, 2011), regulated by microRNA169 and the small peptide uORF1p, and required for nodule meristem persistence and function (Combier *et al.*, 2006, 2008). Here it is demonstrated that the expression of this TF is induced within hours after rhizobial inoculation, significantly before the initiation of nodule organogenesis. Moreover, it is demonstrated that *MtNF-YA1* expression is correlated with the initiation and progression of the symbiotic infection in roots and in nodule tissues, and that a new null mutant allele, *Mtnf-ya1-1*, is strongly affected in IT progression.

## Materials and methods

### Plant growth and bacterial strains

After scarification and surface sterilization as described in Barker *et al.* (2007), *M. truncatula* cultivar A17 Jemalong and *nf-ya1-1* mutant seedlings were germinated and were grown in different conditions depending on the experiment performed. (1) Aeroponic caissons were used when large quantities of infections or nodules or precise synchrony of initial infection steps were required (such as in Figs 2, 4, 5, and Supplementary Figs S1, S2 available at *JXB* online) as described in Barker *et al.* (2007). Plants were inoculated 5 d post-germination with *Sinorhizobium meliloti* GMI6526 as described in Cerri *et al.* (2012). (2) Sepiolite medium-containing pots were used when fully grown, nitrogen-fixing nodules were required (such as in Figs 3, 6, and Supplementary Figs S3–S6). Seedlings were grown in sepiolite (Brenntag SA, St Sulpice, France) supplemented with nitrogen-free caisson medium (Journet *et al.*, 2001). Composite plants with roots expressing the promoter–β-glucuronidase (GUS) or complementation constructs (see below) were obtained using A17 Jemalong seedlings, as described in Boisson-Dernier *et al.* (2001) and inoculated with *S. meliloti* GMI6526 as described in Cerri *et al.* (2012).

### *MtNF-YA1* expression using RNA-seq and qRT-PCR

For the RNA-seq experiment, total RNA was isolated from root tissues using the GeneAll HybridR^+^ kit (GeneAll, Seoul, Korea) and cDNA libraries were synthesized using the TruSeq™ RNA sample preparation kit (Illumina, San Diego, CA, USA) according to the manufacturer’s instructions. The library clusters were sequenced on the Illumina HiSeq2000 sequencer using the TruSeq™ SBS kit v3-HS to generate 100bp single-end sequences.

Quantitative real-time PCR (qRT-PCR) analyses were done following the MIQE Guidelines (Bustin *et al.*, 2009) (http://www.rdml.org/miqe.php) as previously described (Riely *et al.*, 2013). Primers used to quantify *MtNF-YA1* expression were: TTGATAAAGCGTAACAAGCCA (fwd) and TCCTCTTGGTCT ACGCATTG (rev). Two reference genes were used: encoding a 26S proteasome regulatory subunit S5A (Medicago Gene Index: TC108192) and an ubiquitin carrier protein (Medicago Gene Index: TC176441). Similar results were obtained in both cases so data presented here represent relative expression values calculated using TC108192, using primers TGGCAGGAAAGGGTGTTC (fwd) and GCCACCTGAATACCAGCAG (rev).

### DNA constructs

#### Promoter–GUS construct

The promoter of *MtNF-YA1* (a 2.2kb fragment upstream of the first alternative ATG) was amplified from *M. truncatula* A17 Jemalong genomic DNA by PCR using Phusion DNA polymerase (Thermo Fisher Scientific, USA) and recombined into the Gateway^®^ vector pDONRP4-P1R according to the manufacturer’s instructions (Invitrogen). Entry clones for the *GUS* open reading frame (ORF) and the 3′ untranslated region (UTR) of *MtNF-YA1* were obtained in the Gateway vectors pDONR207 and pDONRP2R-P3, respectively. Subsequently entry clones were recombined in the binary vector pK7m34GW (Karimi *et al.*, 2002).

#### Complementation construct

The ORF of *MtNF-YA 1* was amplified from *M. truncatula* nodule cDNA by PCR using Phusion DNA polymerase and recombined in the pDONR207 vector. Entry clones were recombined with the same promoter and 3′UTR construct used for the promoter–GUS construct into pK7m34GW.

Primer sequences used were: attB4 p*MtNF-YA1*, GGGGAC AACTTTGTA TAGAAAAGTTGGTGCCAAATT CAGAGATAC TACTTCC; attB1r p*MtNF-YA1*, GGGGACTGCTTTTTTG TAC AAACTTGATTCAAGTA CTATGTTCTTCTCTATTC; attB1 GUS, GGGGACAAGTTTGT ACAAAAAAGCAGGCTTCATG TTAC GTCCTGTAGAAACCCCAAC; attB2 GUS, GGGGACC ACTTTGTA CAAGAAAGCTGGGTCTTATTGTTT GCCTCCC TGCTGCGGT; attB2r *NF-YA1_*3′UTR, GGGGACAGCTT TCT TGTACAAAGTGGGGGTTTCGATTC AGAAAGGAAACAAG TG; and attB3 *NF-YA1_*3′UTR, GGGGACAACTTTGT ATAA TAAAGTTGGTTACAGAATCCCAA GCCAACATGGTGTTG.

### GUS and β-galactosidase assays

Histochemical staining for GUS activity and bacterial β-galactosidase (β-Gal) activity were performed as described in Cerri *et al.* (2012).

### mRNA *in situ* hybridization

*Medicago truncatula* mature nodules [35 days post-inoculation (dpi)] were fixed, embedded, and sectioned for *in situ* hybridization as described in Valoczi *et al.* (2006). Hybridization was performed at 50 °C using sense (control) and antisense *MtNF-YA1*-specific RNA probes synthesized as follows: a 302bp fragment from the 3′UTR of the gene was amplified from nodule cDNA using the following primers: NF-YA1T7senseFor, *TGTAATACGACT CACTATAGGGC*GTAATATTTA GTAGTATTGTCATT GTCTT TCC; NF-YA1senseRev, AGAGTCTGA AAATAAGAGGTT CT TATAC; NF-YA1antisenseFor, GTAATATTTA GTAGTATTGTC ATT GTCTTTCC; and NF-YA1T7antisenseRev, *TGTAATACGA CTCACTATAGGGC*AGAGTCTGAAAAT AAGAGGTTCTTAT AC. Digoxigenin (DIG)-labelled sense and antisense RNA probes were then obtained by *in vitro* transcription with T7 polymerase using the DIG-RNA labeling kit (Roche Diagnostics). After overnight hybridization, slides were washed in 2× SSC/50% formamide at 50 °C and then treated with RNase A to eliminate non-specific background. Finally the presence of RNA was assessed using alkaline phosphatase–anti-DIG antibodies.

### Acetylene reduction assay

Nitrogen fixation was assayed using nodules from plants grown on sepiolite substrate and harvested 35 dpi with *S. meliloti* strain 2011 (Sm2011). The fresh weight of nodules was measured and their nitrogenase activity was subsequently determined by an acetylene reduction assay (Hardy *et al.*, 1968).

### *Mtnf-ya1-1* mutant description and complementation

The *Mtnf-ya1-1* mutant was identified by a TILLING (Targeting Local Induced Lesions In Genomes) approach within a A17 Jemalong EMS (ethyl methanesulphonate)-mutagenized population. Two sequential backcrosses with A17 were then performed and homozygous lines selected. Root transformation was performed on the *Mtnf-ya1-1* mutant using an empty vector control or the complementation constructs, and kanamycin-selected composite plants (2 weeks) were transferred to the greenhouse (16h light/8h dark, 22 °C, relative humidity 60–70%) on sepiolite substrate imbibed with nitrogen-free caisson medium for 7 d. Transgenic roots were inoculated with 20ml of an Sm2011 suspension (OD_600 nm_=0.05) per plant. Nodule development was observed at 21 dpi. Three biological experiments were performed with a minimum of 30 independent plants.

### Anti-MtNF-YA1 antibody production and western blot analysis

Anti-NF-YA1 polyclonal antibodies were raised in rabbit by Eurogentec (http://www.eurogentec.be) against two peptides at the N-terminus of MtNF-YA1; namely, ^1^MAMQPVYLKEHEGNV^15^ and ^64^APSKNLVRGVEQLFD^78^. To analyse protein expression, wild-type (WT) and *MtMtnf-ya1-1* mutant plants were grown aeroponically, inoculated with *S. meliloti*, and nodules were then harvested and ground in liquid nitrogen. An aliquot of 50–100mg of powder was then resuspended in 100 μl of extraction buffer [50mM TRIS-HCl at pH 7.4, 150mM NaCl, 10% glycerol (v/v), 1mM dithiothreitol (DTT), 1mM phenylmethylsulphonyl fluoride (PMSF), and 1% plant protease inhibitor cocktail (Sigma)] and centrifuged at 10 000 *g* for 10min at 4 °C. The protein concentration in the supernatant was determined with the Bradford protein assay kit (Bio-Rad), using bovine serum albumin (BSA) as a standard. A 50 μg aliquot of total protein was separated on NuPage 10% BIS-TRIS gels (Invitrogen) according to the manufacturer’s instructions and transferred onto Protran BA85 nitrocellulose membranes (Schleicher & Schuell) by wet electroblotting (Mini-Protean II system; Bio-Rad). For detection of MtNF-YA1, blots were incubated with anti-NF-YA1 polyclonal antibodies (1:10 000 dilution). After incubation with the secondary antibody (anti-rabbit IgG–peroxidase, 1:15 000; Sigma), bands were visualized using the ECL Plus kit (Amersham Pharmacia Biotech) under standard conditions.

### Microscopic observations

Root or nodule tissues were observed with a light microscope (Axiophot, Carl Zeiss, Oberkochen, Germany) and photographed using an M-1300-HS CCD camera (Princeton Instruments, Evry, France). Green fluorescent protein (GFP)-expressing rhizobia in ITs were imaged in roots of *Mtnf-ya1-1* or *sunn-2* supernodulant plants as previously described (Cerri *et al.*, 2012). The images were processed using the Leica confocal and Fiji softwares (Schindelin *et al.*, 2012), and maximal projections of selected planes of a *z*-stack are shown.

### Electron microscopy

Nodules were harvested 4 weeks following inoculation, fixed with a solution of 2.5% glutaraldehyde in 0.1M potassium phosphate (pH 7.2) under vacuum, and post-fixed with a 2% OsO4 aqueous solution. The histological organization of nodules was observed on semi-thin sections (1 μm) of Epon-embedded nodules, stained by 0.2% toluidine blue. Observations were performed using bright field microscopy with AxioPlan Imaging (Zeiss, Jena, Germany). Ultrastructure was studied on ultrathin sections (70nm, Ultracut, Reichert, Germany) of Epon-embedded nodules stained with uranyl acetate and observed with a Hitachi HT7700 electron microscope (Hitachi, Tokyo, Japan). Measurements of IT wall thickness were performed using the ImageJ software (Perkin Elmer). Four to five measurements were made on the entire circumference of each IT measured; oblique sections in which the thickness of walls was clearly an artefact were avoided.

### Statistical analysis

Analysis of variance (ANOVA) modellization followed by a multiple comparison test (Yandell, 1997) was applied on quantitative expression and nodule number data using a *P*-value threshold of 0.05.

A Wilcoxon rank sum test (Hollander and Wolfe, 1973) was used to compare the number of ITs and cell wall width between the A17 WT and the *MtNF-YA1-1* mutant.

## Results

### *MtNF-YA1* expression is associated with the symbiotic infection process

**Fig. 1.**
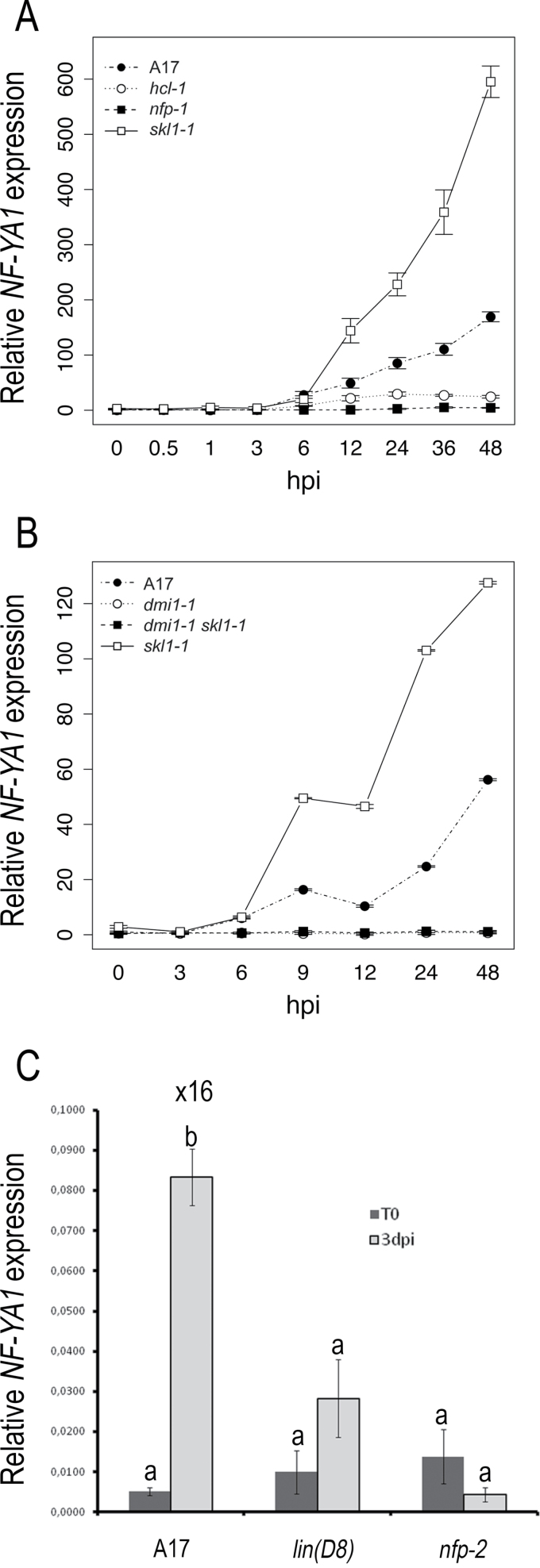
Expression analysis of *MtNF-YA1* during early stages of the symbiotic interaction between *Medicago truncatula* and *Sinorhizobium meliloti*, using qRT-PCR and RNA-seq. (A) Relative expression of *MtNF-YA1* in *M. truncatula* A17, *lyk3* (*hcl-1*), *nfp* (*nfp-1*), and *skl* roots at various times after inoculation [hours post-inoculation (hpi)] as estimated by RNA-seq. Values represent the average of four biological replicates; bars represent standard errors. The graph was generated using the sciplot package in R (cran.r-project.org/package=sciplot). (B) Relative expression of *MtNF-YA1* in *M. truncatula* A17, *dmi1* (*dmi1-1*), *skl*, and *dmi1 skl* roots at various times after inoculation (hpi) as estimated by qRT-PCR. Values represent the average of three technical and three biological replicates; bars represent standard errors. The graph was generated using the sciplot package in R. (C) qRT-PCR analysis of *MtNF-YA1* expression in entire roots, before inoculation (T0, dark grey boxes) and 3 days post-inoculation (3 dpi, light grey boxes), in three different genetic backgrounds, WT A17, the *lumpy infection* mutant (*lin*, allele D8), and the *nod-factor perception* mutant (*nfp*, allele *nfp-2*). Data shown are the average of three biological repeats. Bars represent standard errors. An ANOVA modelling followed by a multiple comparison test showed that using a *P*-value of 0.05, only the 3 dpi value (b) for WT A17 was statistically different from all the other values (a).

In previous reports, it was shown that *MtNF-YA1* (previously called *HAP2-1*) is highly and specifically expressed in mature nodules, specifically in non-infected meristematic cells and in cells of the infection zone (El Yahyaoui *et al.*, 2004; Combier *et al.*, 2006). To determine if *MtNF-YA1* is expressed during earlier phases of the symbiotic interaction, especially during the initial stages of the root infection process, a detailed expression time course was conducted using RNA-seq. As shown in Fig. 1A, *MtNF-YA1* was strongly up-regulated in whole roots as early as 6h after inoculation, preceding infection by *S. meliloti* which is first evident at 48h. *MtNF-YA1* expression levels rose continuously from 6h to 48h post-inoculation, throughout the phases of pre-infection, infection, and early nodule morphogenesis. Ethylene-insensitive *ein2* (*skl1-1*) mutants of *M. truncatula* show a dramatic increase in the number of sustained infections (Penmetsa *et al.*, 2008) and a corresponding substantial up-regulation of *MtNF-YA1* was observed in *skl* plants relative to WT *M. truncatula* A17 (Penmetsa and Cook, 1997). In contrast, levels of expression remained unchanged in the NF-insensitive *nfp-1* (C31) mutant (Ben Amor *et al.*, 2003), and induction was significantly impaired in the *lyk3* mutant allele *hcl-1* (B56) that is blocked for bacterial entry and impaired for NF-induced gene expression (Limpens *et al.*, 2003; Smit *et al.*, 2007). To confirm this early induction and to assess the relationship between NF and ethylene perception pathways in *MtNF-YA1* expression, qRT-PCR was used to quantify expression in four *M. truncatula* genotypes: A17, *skl1-1*, the nod-minus mutant *dmi1-1* (C71), and a double mutant *dmi1-1 skl1-1*. As shown in Fig. 1B, qRT-PCR revealed the expected strong and early up-regulation of *MtNF-YA1* in WT A17 and its superinduction in *skl*. *MtNF-YA1* induction was not detected in either *dmi1-1* or the double mutant *dmi1-1 skl*. Taken together, these results demonstrate that *MtNF-YA1* expression is up-regulated early (6h) upon rhizobial inoculation, that this up-regulation is NF dependent, that superinduction in *skl* is dependent on the NF pathway, and that there are temporal and quantitative correlations between rhizobial infection and the level of *MtNF-YA1* expression.

A related experiment, focusing on later time points, was conducted using qRT-PCR to evaluate expression in non-inoculated roots and at 3 dpi, a stage at which, in the hydroponic culture system used here, numerous ITs are found in the epidermis and outer cortical layers and cortical cell divisions are evident in the inner cortex. As shown in Fig. 1C, strong induction of *MtNF-YA1* expression (16-fold) was observed in WT roots at 3 dpi. Parallel analyses were conducted with two *M. truncatula* mutant lines, namely the NF- insensitive LysM RLK mutant *nfp* (*nfp-2*) and the nodulation-defective mutant *lin*, which encodes a U-box/WD40 protein. *lin* plants exhibited a strong reduction in *MtNF-YA1* induction relative to the WT, which correlates with the 4-fold reduction in the number of infections in *lin*, all of which arrest in the root epidermis, and nodule primordia that initiate but then fail to develop a persistent meristem (Kuppusamy *et al.*, 2004; Kiss *et al.*, 2009). In contrast, as determined above, *nfp* mutants failed to induce *MtNF-YA1* expression upon *S. meliloti* inoculation.

To describe *MtNF-YA1* expression in greater detail, particularly during early infection stages, a reporter construct was produced bearing 2.2kb of the *MtNF-YA1* promoter sequence fused to the GUS gene (p*MtNF-YA1-GUS*). This promoter was functionally validated by complementation of the *Mtnf-ya1-1* mutant, as described below (Fig. 3E). The 3′UTR of *MtNF-YA1* has been shown previously (Combier *et al.*, 2006) to contain a recognition site for MIR169, which is important for its proper temporal and spatial regulation, and thus this 3′UTR sequence was also included in the reporter construct. p*MtNF-YA1-GUS* was transformed into *M. truncatula* roots using *Agrobacterium rhizogenes-*mediated root transformation. *MtNF-YA1* expression was weak (barely detectable) in non-inoculated roots, with the notable exceptions of the main root tips and early developing lateral roots (Supplementary Fig. S1A–D at *JXB* online).

**Fig. 2.**
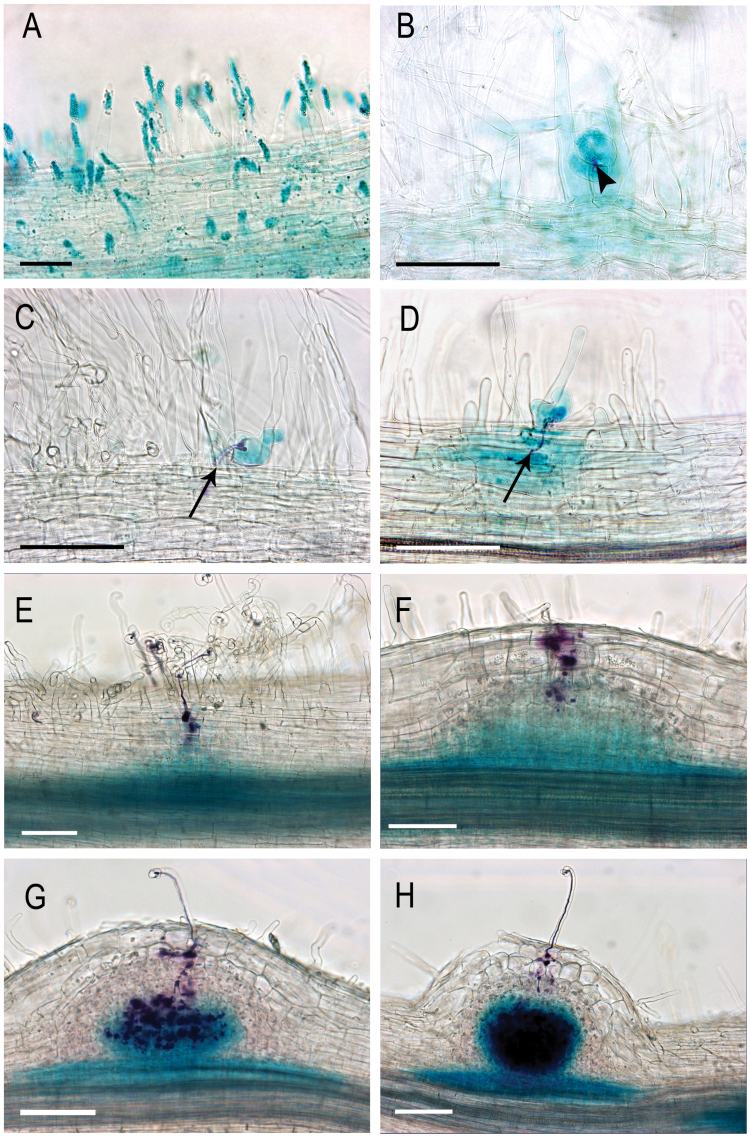
Expression analysis of *MtNF-YA1* during early stages of the symbiotic interaction between *Medicago truncatula* and *Sinorhizobium meliloti* using a promoter–GUS reporter gene. Double staining using Magenta-Gal and X-Gluc allowing the visualization of the infecting *S. meliloti* in purple and *MtNF-YA1* expression in blue. (A) Root hairs in the pre-infection zone, 24hpi. (B and C) Microcolony (black arrowhead, B) in the centre of a curled root hair 48hpi expressing *MtNF-YA1*. (C) Infected root hair crossed by an infection thread (IT) (black arrow, C) and expressing *MtNF-YA1*, 48h post-inoculation. (D and E) ITs (black arrows) that are reaching the cortex are shown; 72hpi, *MtNF-YA1* expression is mainly found associated with infected root hairs or cortical cells or cells in contact with the infected cells. (E) Note also the expression of *MtNF-YA1* ahead of the IT in dividing cells of the inner cortex. (F) At 72hpi, *MtNF-YA1* expression is shown in a nodule primordium facing an infection site, and around ITs reaching the inner cortical layers. (G and H) At 96hpi, *MtNF-YA1* is expressed strongly in the central part of developing nodules in cells in contact with ITs or released bacteria. Bars represent 100 μm.

As already shown in Fig. 1, p*MtNF-YA1-GUS* expression was induced well in advance of *S. meliloti* infection, which in the system used here was not evident until 48h post-inoculation. Root hairs of the pre-infection zone of transgenic roots strongly and specifically expressed p*MtNF-YA1-GUS* by 24h after inoculation (Fig. 2A). While this up-regulation was observed in many root hairs of the pre-infection zone, p*MtNF-YA1-GUS* expression was predominantly expressed in curled and infected root hairs during subsequent stages. *MtNF-YA1* expression was observed in individual root hairs as soon as they curled, forming the typical ‘shepherd’s crook’ and entrapping a microcolony of *S. meliloti* at 24–48h post-inoculation (Fig. 2B). This tight association between *MtNF-YA1* expression and the infection process was maintained throughout IT growth, first through the epidermis (Fig. 2C), subsequently through the different cortical layers (Fig. 2D, E), and finally during the initial stages of nodule development (Fig. 2F–H). Within young nodules, expression was primarily in the region of IT growth (Fig. 2G) and in cells after release into symbiosomes (Fig. 2H), while in fully differentiated nodules expression was confined to the apical region (Supplementary Fig. S2G, H at *JXB* online). *MtNF-YA1* expression was also observed in dividing cells of nodule primordia at a distance from infecting bacteria (Fig. 2E, F). The up-regulation of *MtNF-YA1* expression during rhizobial infection, however, appeared relatively transient and linked to initial phases of infection, as can be observed in Fig. 2E–H, in which the epidermal and cortical cells crossed by ITs no longer express *MtNF-YA1*. During subsequent steps of nodule development, *MtNF-YA1* was expressed strongly and uniformly in the central part of growing nodules (Fig. 2G, H; Supplementary Fig. S4), before gradually becoming more expressed in the apical zone as the different nodule tissues differentiated (Supplementary Fig. 2G, H). Expression at the base of young developing nodules was, in addition, consistently observed across experiments but faded in mature nodules. This further illustrates the expression of *MtNF-YA1* in internal tissues activated by rhizobia. In mature nodules, p*MtNF-YA1-GUS* expression was restricted to the distal end. Expression followed a gradient of decreasing intensity from the meristematic zone down to the proximal part of the infection zone (Supplementary Figs S2, S3). Non-radioactive mRNA *in situ* hybridization analyses of mature nodules confirmed, with higher cellular resolution, the localization of *MtNF-YA1* mRNA within the meristem and infection zones (i.e. zones 1 and 2; Supplementary Fig. S3), as observed previously in Combier *et al.* (2006).

### Identification and characterization of the *Mtnf-ya1-1* null mutant

**Fig. 3.**
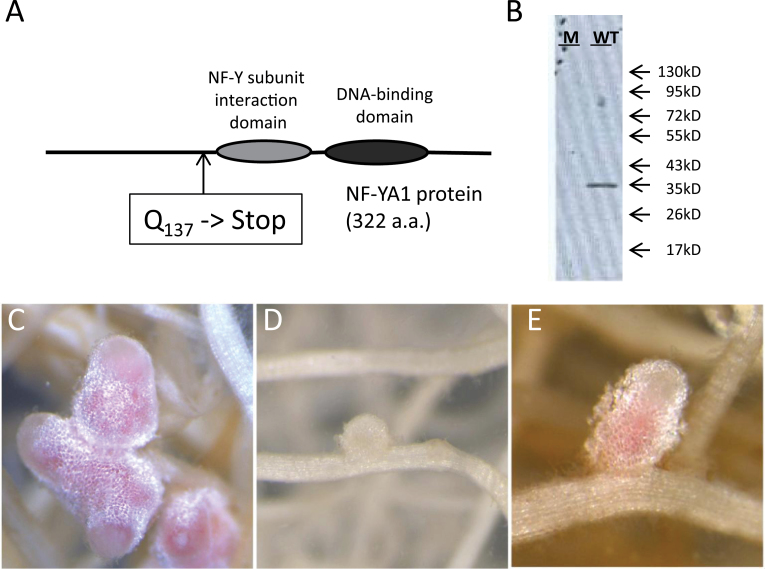
*Mtnf-ya1-1* mutant description and complementation. (A) Schematic view of the non-synonymous mutation in *Mtnf-ya1-1* leading to a premature stop codon at amino acid position 137. (B) Western blot analysis of wild-type (WT) and *Mtnf-ya1-1* mutant (M) nodule proteins using an anti-NF-YA1 antibody and showing the absence of MtNF-YA1 protein (36kDa) as well as the absence of significant amounts of a shorter, truncated version of the protein in *Mtnf-ya1-1*. (C–E) Complementation experiment. A17 WT plants (C) and mutant *Mtnf-ya1-1* plants (D, E) were transformed with empty vector control (C, D) or with *MtNF-YA1* expressed under the control of its own promoter (E) using *Agrobacterium rhizogenes* transformation. Composite plants were subsequently inoculated with *Sinorhizobium meliloti* and roots were harvested for examination at 21 dpi.

To assess the function of *MtNF-YA1*, an EMS mutant population of *M. truncatula* was screened using the TILLING approach (Henikoff *et al.*, 2004). Among the alleles obtained, a non-synonymous mutation converting the glutamine in position 137 into a premature stop codon was identified and the corresponding mutant was named *Mtnf-ya1-1*. The stop codon in *Mtnf-ya1-1* occurs upstream of the two main conserved regions found in NF-YA proteins across kingdoms, and which have been shown to be essential for the interaction of NF-YA with the NF-YB and NF-YC components and for binding to CCAAT boxes within promoters, respectively (Fig. 3A) (Mantovani, 1999). Western blot experiments in nodule tissues using anti-peptide antibodies raised against the N-terminal part of the protein showed that neither the 322 amino acid (36.4kDa) MtNF-YA1 full-length protein nor the 136 amino acid (15.2kDa) putative truncated protein that should in theory be produced in the mutant was detectable in the *Mtnf-ya1-1* mutant. The absence of the truncated protein is possibly a consequence of nonsense-mediated mRNA decay (NMD) (Kervestin and Jacobson, 2012) (Fig. 3B). The *Mtnf-ya1-1* mutant thus appears to be a null mutant allele of *MtNF-YA1* and, as shown below, this mutant is strongly affected in nodule development, comparable with previous results obtained with RNA interference (RNAi) transcript suppression (Combier *et al.*, 2006). In order to confirm that the symbiotic phenotypes observed in the *Mtnf-ya1-1* mutant were truly due to the absence of MtNF-YA1, *Mtnf-ya1-1* plants were complemented with ectopic *MtNF-YA1*, introduced by means of *A. rhizogenes*, and expressed under the same native promoter region used for the promoter–GUS experiment above. Complemented plants developed fully grown pink nodules (Fig. 3E) equivalent to those observed in A17 roots (Fig. 3C) and unlike the small and white nodules characteristic of the *Mtnf-ya1-1* mutant (Fig. 3D).

**Fig. 4.**
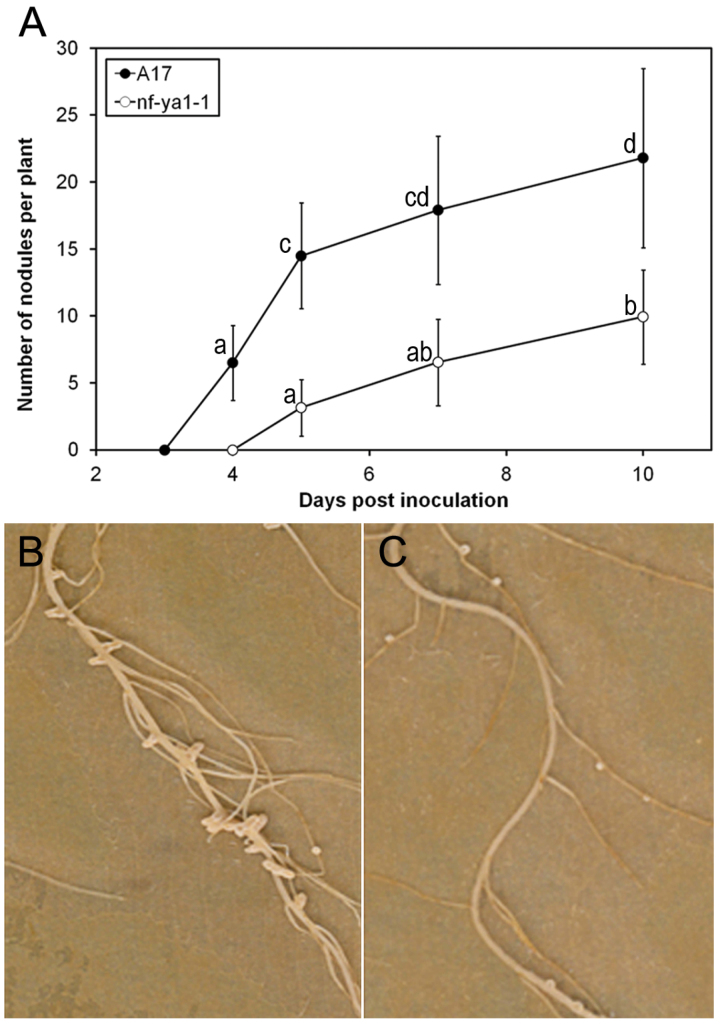
Nodule development in A17 WT and in the *Mtnf-ya1-1* mutant. (A) WT and *Mtnf-ya1-1* plants were grown aeroponically, and inoculated with *Sinorhizobium meliloti*. The roots were then harvested and the number of nodules per plants was counted 4, 5, 7, and 10 days post-inoculation. Each point is the average of 13 plants. An ANOVA modelling followed by a multiple comparison using a *P*-value of 0.05 was applied to these data. Letters from a to d illustrate the different groups of statistically different data points. (B and C) Picture of nodulated root systems, 21 dpi in WT (B) and *Mtnf-ya1-1* (C) roots. Note the reduced number and size of mutant nodules as well as their different position, more on side roots and less on main roots, in the *Mtnf-ya1-1* mutant compared with the WT.

As shown in Fig. 4, *Mtnf-ya1-1* mutants have delayed and reduced nodule development. Under the growth conditions used here, nodules emerge between 3 and 4 dpi in WT A17, but were not evident until 5 dpi in *Mtnf-ya1-1* plants (Fig. 4A). In addition to a delay in the initiation of nodule development, the number of nodules formed in *Mtnf-ya1-1* mutants was significantly reduced compared with WT plants (Fig. 4). This was most obvious at 5 dpi when on average 4.6-fold fewer nodules had formed in the *Mtnf-ya1-1* mutant compared with A17; this difference was 2.4-fold, yet significant, at 10 dpi (Fig. 4A). No effect on root length was observed in the *Mtnf-ya1-1* mutants (Supplementary Fig. S4 at *JXB* online), but the distribution of nodules on the root system was different between A17 and the mutant, with most nodules present on the main root in A17 and preferentially on lateral roots in *Mtnf-ya1-1* mutants (Fig. 4B, C). Moreover, *Mtnf-ya1-1* nodule morphology was strongly affected (Supplementary Fig. S5A) (Vasse *et al.*, 1990) and nitrogen fixation was strongly altered in these mutant nodules; indeed *Mtnf-ya1-1* nodules were not fully fix^–^ but fixed nitrogen at rates significantly below those of the WT when compared on a fresh weight basis (Supplementary Fig. S5B). In *Mtnf-ya1-1*, light and electron microscopy revealed reduced symbiosome formation, though in the few cases when bacterial release occurred differentiated bacteroids could be observed (Supplementary Fig. S6).

### *MtNF-YA1* controls rhizobial infection

**Fig. 5.**
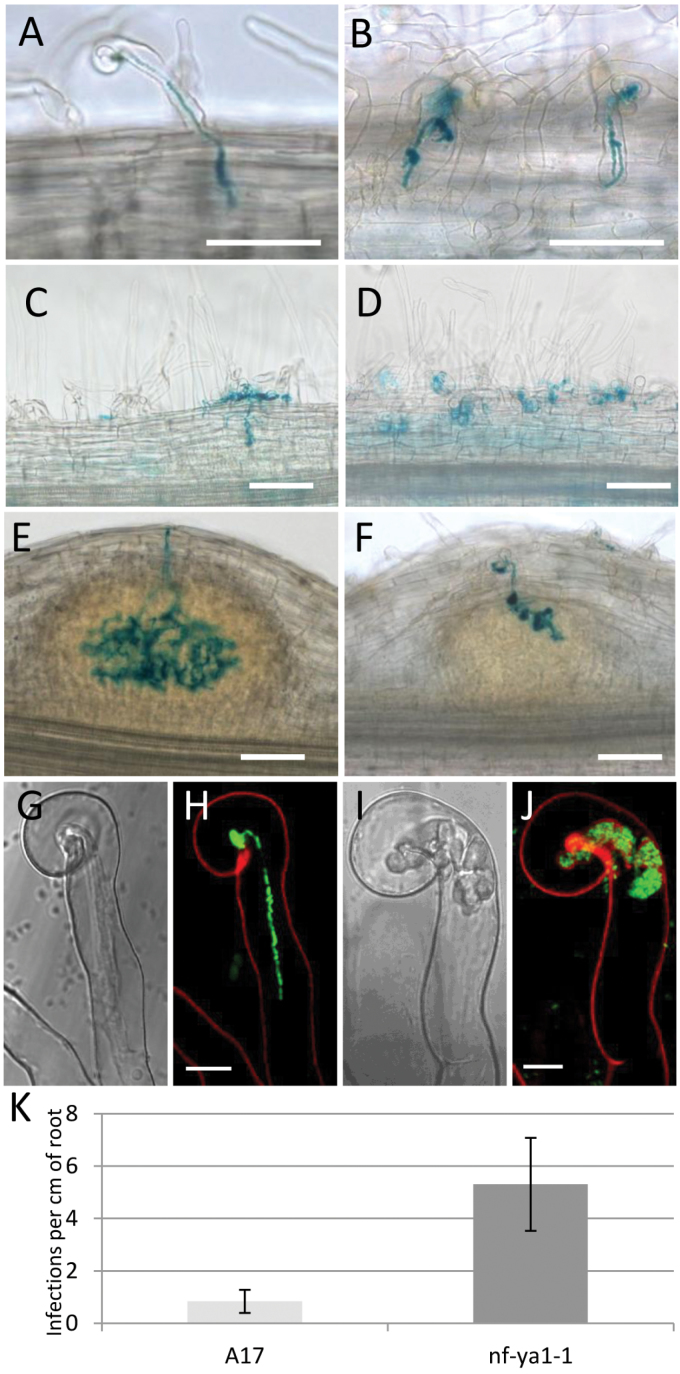
Infection phenotype of the *Mtnf-ya1-1* mutant during early stages of the symbiotic interaction between *Medicago truncatula* and *Sinorhizobium meliloti*. The presence of bacteria in blue is visualized by a β-Gal assay. Light microscopy pictures of WT (A, C, E) and *Mtnf-ya1-1* mutant (B, D, F) roots inoculated by *S. meliloti.* (A and B) Epidermal infection threads (ITs) observed 48hpi; note the swollen and branched aspect of mutant ITs. (C and D) ITs observed 72hpi; note the difference in progression of ITs between the WT and mutant. (E and F) ITs observed 96hpi. Note again the swollen and bulbous aspect of mutant ITs also in the cortex and the delay in IT progression and nodule organogenesis. (G–J) Brightfield (G, I) and confocal images (H, J) of WT (G, H) and *Mtnf-ya1-1* mutant (I, J), root hairs, 48hpi by *S. meliloti* and illustrating the abnormal swollen and bulbous phenotype of ITs in mutant root hairs. Fluorescent bacteria can be observed in green, while the red colour is the autofluorescence of the wall, in confocal images H and J. White bars represent 100 μm for A–F and 10 μm for G–J. (K) Quantification of infection points in WT (A17) and *Mtnf-ya1-1* mutant roots. Plants were grown aeroponically, inoculated with *S. meliloti*, and harvested 5 dpi; bacterial infections were revealed by β-Gal assays, counted under the light microscope, and this number was reported relative to the length of the root. While most infections had progressed to the cortex in WT roots at this stage, most infections were present in the epidermis in *Mtnf-ya1-1* mutant roots. Note the 6.3-fold higher number of infections in the roots of mutants compared with the WT. Data presented are the average obtained for 20 root systems. A Wilcoxon rank sum test showed that the two data sets are statistically different with a highly significant *P*-value of 1.45e-11.

The expression of *MtNF-YA1* during early steps of the symbiotic interaction with *S. meliloti*, together with the observed delay in nodule development in the *Mtnf-ya1-1* mutant, led to examination of whether this mutant was also affected in symbiotic infection. Using an *S. meliloti* strain constitutively expressing the *lacZ* reporter gene, the infection process was compared in WT A17 and *Mtnf-ya1-1* mutant roots. Progression of ITs was clearly delayed and abnormal in the *Mtnf-ya1-1* mutant, during all symbiotic steps investigated. Indeed, compared with WT ITs that mainly progress through root hairs by forming a thin and smooth tubular structure (Fig. 5A), ITs in the mutant line appear thicker, bulbous, and branched, with frequent signs of arrested growth at the epidermal layer and often multiple ITs emerging from the bulbs (Fig. 5B). In most cases, these ITs did not progress towards the cortical layers of the roots but stayed arrested in the epidermal layer, either at the microcolony stage or mainly inside root hairs (Fig. 5C, D). In contrast, infection points (infected root hairs and more rarely microcolonies) appeared much more numerous in roots of the *Mtnf-ya1-1* mutant than in the WT (Fig. 5A–D). As shown in Fig. 5K for 5 dpi roots, a significant 6.3-fold increase in the number of infection points per cm of root was observed in *Mtnf-ya1-1* compared with A17, confirming the observation of more frequent arrested infection events in the mutant. In addition, by 3 dpi, most ITs had reached the cortex in WT A17, and cortical cells situated under the cortical threads had started dividing (Fig. 5C). Comparable analyses in *Mtnf-ya1-1* failed to detect signs of cortical division under epidermal ITs. Occasionally, ITs reached the outer cortex in *Mtnf-ya1-1*, but they possessed the same abnormal swelling phenotype observed in less progressed infections, and nodule initiation and development were significantly retarded compared with A17 (Fig. 5E, F).

**Fig. 6.**
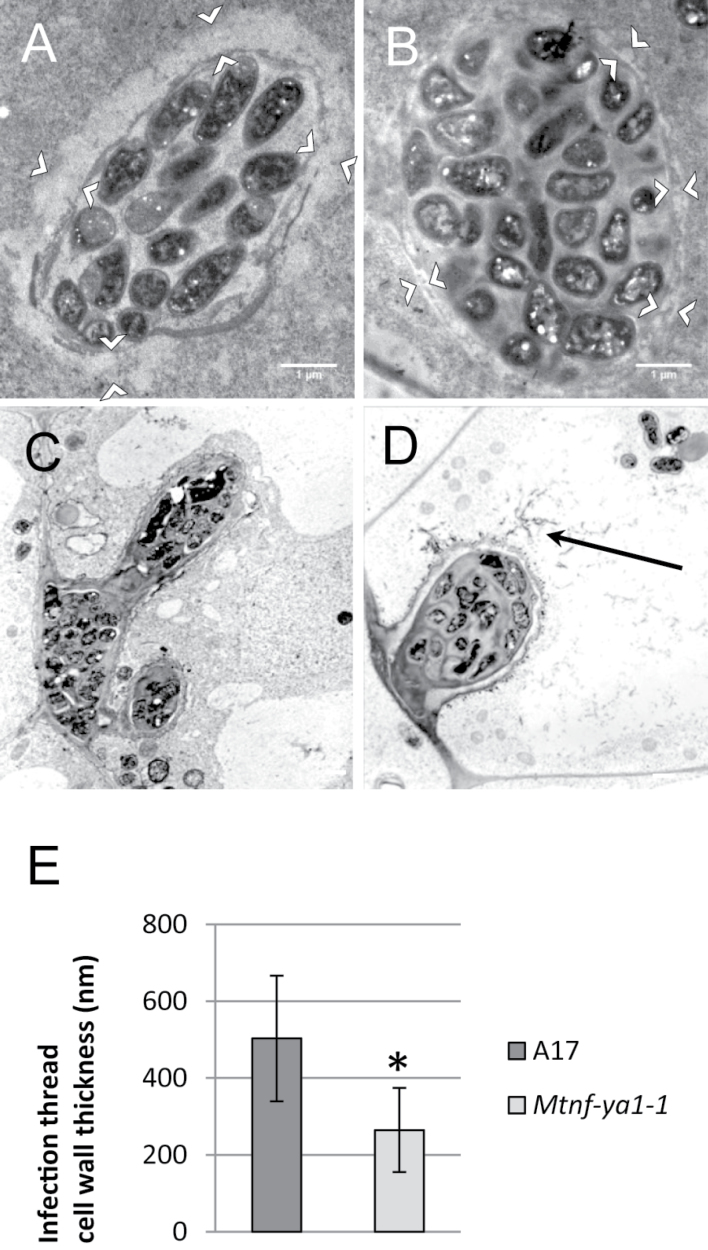
Analysis of infection thread (IT) ultrastructure using transmission electron microscopy. (A and B) Cross-section through an IT of the infection zone of a nodule 21 dpi in WT A17 (A) and in the *Mtnf-ya1-1* mutant (B). Note the clear difference in IT wall thickness which is less in the mutant compared with the WT (white arrowheads). (C and D) Section through an IT of the fixation zone of a nodule 21 dpi in WT A17 (C) and in the *Mtnf-ya1-1* mutant (D). Note that the IT wall is not only thinner in the mutant but also appears less coherent and more fragile, with fibres that come off the wall into the cytoplasm of the infected host cell (black arrow). (E) Quantification of IT wall thickness in the infection zone of WT A17 nodules (dark grey) and *Mtnf-ya1-1* mutant nodules (light grey). The data shown are the average of 24 measurements. A Wilcoxon rank sum test showed that the two data sets are statistically different with a highly significant *P*-value of 8.86e-08.

Confocal microscopy was used to examine IT morphology further in the *Mtnf-ya1-1* mutant by means of GFP-tagged *S. meliloti* as described in Fournier *et al.* (2008). As shown in Fig. 5G–J, ITs in the WT were thin and straight, with a single column of infecting bacteria at early stages, whereas *Mtnf-ya1-1* mutant ITs appeared as thickened sac-like swellings, densely filled with bacteria but without polarity, connected by thin regions of apparent transient polar growth. The ultrastructure of WT and mutant ITs was then compared using transmission electron microscopy. Transverse sections through an IT within infection zone 2 of a 10-day-old nodule revealed a significantly thicker IT cell wall in WT compared with mutant plants (Fig. 6A, B; Supplementary Fig. S6 at *JXB* online). IT cell wall thickness was measured in 24 sections of both the WT and mutant (Fig. 6E), revealing a statistically significant 2-fold decrease in wall thickness of mutant ITs. Moreover, the ultrastructure of the mutant IT wall appeared less coherent and more fragile, with fibres that protrude from the wall into the cytoplasm of the infected host cell (Fig. 6C, D).

## Discussion

*MtNF-YA1* was first described as a symbiosis-up-regulated TF of the CCAAT box-binding family expressed in the meristematic zone of mature nodules and thought to play a role in nodule meristem functioning (Combier *et al.*, 2006). In this study, using detailed expression analysis and functional data coming from a new null mutant allele, *Mtnf-ya1-1*, evidence is presented for an additional role for *MtNF-YA1* during infection of *M. truncatula* by *S. meliloti*.

The present RNA-seq, qRT-PCR, and promoter–GUS expression data document that *MtNF-YA1* expression is up-regulated by *S. meliloti* as early as 6h post-inoculation, but most substantially between 12h and 48h and 3 dpi. Thus *MtNF-YA1* is among the earliest known rhizobium-induced symbiotic TFs, consistent with a role during early steps of the symbiotic interaction. During these initial phases, *MtNF-YA1* is up-regulated specifically in root hairs of the susceptible zone of roots, which are the first root cells known to respond to rhizobial signals and rhizobia. The fact that this up-regulation is not observed in the *nfp* and *dmi1* mutants demonstrates that induction of *MtNF-YA1* expression depends on NF perception and signal transduction machinery. Interestingly, *MtNF-YA1* is also induced, albeit at low levels, in the *lyk3* mutant *hcl-1*, a genotype that lacks infection but exhibits strong NF-dependent root hair deformation responses (Ben Amor *et al.*, 2003). Taken together, these expression data support the notion that *MtNF-YA1* may function prior to infection in WT plants, and indeed the delayed infection and nodule development phenotypes of the *Mtnf-ya1-1* mutant are consistent with this idea. Thus it is speculated that *MtNF-YA1* is important during pre-infection phases, potentially in preparation for and/or initiation of infection.

Beyond this potential early role during infection initiation, *MtNF-YA1* transcript increases in abundance throughout the early interaction, despite the fact that the domain of *MtNF-YA1* expression becomes increasingly focused and ultimately concentrated in root hairs containing infection and then in nodules. The kinetics of this expression and the tight correlation between rhizobial infection and *MtNF-YA1* expression in infected cells suggest a role for this TF during rhizobial infection, potentially as an extension of the pre-infection role postulated above. Thus throughout early symbiotic development, from the microcolony stage, through root hair penetration and cortical cell invasion, to bacterial release in the nodule infection zone, *MtNF-YA1* is continually expressed in conjunction with bacterial infection. The strongly reduced up-regulation of *MtNF-YA1* in *lin*, a mutant that has fewer infections and is blocked in IT progression but not in rhizobial entry nor the formation of nodule primordia, further suggests a role for *MtNF-YA1* during rhizobial infection. This correlation between levels of *MtNF-YA1* expression and the extent of rhizobial infection is extended by the superinduction of *MtNF-YA1* in the hyperinfected mutant *skl*. The relatively transient expression pattern upon inoculation of *MtNF-YA1* provides additional evidence for a role for this TF in initial phases of infection. However, the strongest evidence for an infection-related function of *MtNF-YA1* comes from the marked infection phenotype of the *Mtnf-ya1-1* null mutant allele. In particular, a 6-fold increase was observed in the average number of ITs in root hairs of *Mtnf-ya1-1* compared with the WT, but IT morphology was abnormal and IT growth was typically arrested. Such an increase in unsuccessful epidermal infections in mutants controlling rhizobial infection has been observed before, for example in *Vapyrin* mutants (Murray *et al.*, 2011). Indeed, most of the ITs observed in the *Mtnf-ya1-1* mutant both in the epidermis and in cortical layers, when ITs manage to reach there, were swollen and showed signs of frequent arrest and/or erratic orientation. These observations implicate *MtNF-YA1* in control of IT progression, while other aspects of infection such as bacterial release from ITs into symbiosomes appear rather normal (though reduced in frequency). Instead, ultrastructural analyses revealed that the cell wall of ITs is altered in the *Mtnf-ya1-1* mutant, with thinner and friable IT walls in both the epidermis and cortex. Modified IT cell wall integrity could explain the bulbous and erratic IT growth phenotypes observed in *Mtnf-ya1-1*, with the IT being too fragile to contain growing bacteria properly.

Little is known about the composition of IT cell walls. Immunocytochemistry suggests that the IT walls in pea (*Pisum sativum*) closely resemble primary cell walls of the surrounding cells (Rae *et al.*, 1992), at least in mature ITs, but no study describing the precise carbohydrate or protein composition of this compartment, especially during its formation, has been performed. Nevertheless, plant cell wall remodelling is integral to early events of the legume–rhizobium symbiosis (Brewin, 2004). Indeed, many nodule up-regulated genes are specialized members of gene families encoding cell wall structural proteins, especially proline-rich, arabinogalactan proteins, while others encode enzymes implicated in cell wall synthesis or modification (Brewin, 2004; El Yahyaoui *et al.*, 2004). Despite this wealth of expression data, genetic evidence demonstrating a function for such genes is lacking and there is relatively little known about genes that control IT initiation and progression. Recently Xie *et al.* (2012) documented a role for *L. japonicus.* nodule pectate lyase (NPL) in the initiation of rhizobial infection, but it is uncertain whether this enzyme participates directly in IT formation. Given the present hypothesis that *MtNF-YA1* controls rhizobial infection via effects on IT structure, it is of particular interest to explore the expression of cell wall remodelling enzymes in the *Mtnf-ya1* mutant background.

Nodule organogenesis and rhizobial infection can be separated genetically, as shown by the *L. japonicus hyperinfected* mutant (*hit1*) in which large numbers of ITs can be observed in the cortex in the absence of cortical cell division (Murray *et al.*, 2007), and also by the gain-of-function mutations in *CCamK* (*DMI3*) (Gleason *et al.*, 2006; Tirichine *et al.*, 2006) and in the cytokinin receptor gene *LHK1* (Murray *et al.*, 2007; Tirichine *et al.*, 2007), in which spontaneous nodulation can be triggered in the absence of infecting rhizobia. Conversely, several reports show the interdependence of the two genetic programmes. In pea the characterization of the *sym33* mutant indicates that successful infection is required for full elaboration of the indeterminate nodule meristem (Voroshilova *et al.*, 2009). In *L. japonicus*, reciprocal crosses between the two gain-of-function alleles *snf1* and *snf2*, leading to spontaneous nodulation, and several loss-of-function mutants blocked in the infection process reveal the existence of cross-talk between pathways for organogenesis and infection and suggest that activated CCamK (Ca^2+^/calmodulin-dependent kinase) and Cyclops mediate cross-pathway signalling between the two pathways (Hayashi *et al.*, 2010; Madsen *et al.*, 2010). More recently, in *M. truncatula* the analysis of the *lin-4* mutant allele, which is blocked in IT initiation at the microcolony stage and forms abnormal nodules with centralized vasculature, suggests that IT initiation is required for normal nodule development (Guan *et al.*, 2013). Here it is shown that in addition to being strongly expressed in the apical tissues of mature nodules and regulating nodule meristem function and thus nodule organogenesis (Combier *et al.*, 2006, 2008), *MtNF-YA1* is also expressed during, and controls, rhizobial infection in *Medicago*, both during initial steps of root infection and later during the infection of nodule tissues.

Pinpointing the primary effect of a mutation in the *MtNF-YA1* gene is difficult, especially given the potential of TFs to regulate multiple genes and processes. Thus a perturbed infection process could impede cortical cell divisions and nodule meristem formation, or vice versa, or *MtNF-YA1* could directly impact both phenomena as its expression pattern suggests. Indeed, such a dual role has been previously shown for other symbiotic genes. Weak alleles or RNAi lines only partially reducing gene expression have shown, for example, that *NFP*, *LYK3*, and *DMI2* also play a role during rhizobial infection beyond their role in initiating symbiosis, though both early and late phenotypes of these genes could derive from their impacts on NF perception and signal transduction (Limpens *et al.*, 2005; Arrighi *et al.*, 2007; Smit *et al.*, 2007). This is also the case for other symbiotic TFs such as *MtNIN*, which was shown to be required for autoactive CCamK-induced nodule organogenesis (Marsh *et al.*, 2007). However, *nin* mutants are blocked in infection initiation (Schauser *et al.*, 1999) and *NIN* has also been shown to control the symbiotic expression of *NPL* that is required for rhizobial infection in *L. japonicus*. Furthermore, the GRAS-type TFs NSP1 and NSP2 also both control infection and nodule organogenesis (Mitra *et al.*, 2004b; Kalo *et al.*, 2005; Smit *et al.*, 2005). NSP1 and NSP2 have been shown to dimerize and to bind a promoter region called the infection box in the *MtEnod11* promoter, but also to activate the expression of ERN1, another TF involved in both rhizobial infection and nodule organogenesis (Andriankaja *et al.*, 2007; Middleton *et al.*, 2007; Cerri *et al.*, 2012). Many of these TFs probably interact and influence each other’s expression in complex regulatory circuits. While it has recently been shown in *L. japonicus* that *NIN* directly controls the expression of *NF-YA1* (Soyano *et al.*, 2013), the relationship between *MtNF-YA1* and *NSP1* and *NSP2* remains to be established, and unravelling these pathways should lead to a better understanding of the cross-talk between nodule development and infection.

Compared with the drastic infection phenotypes of *ern1*, *nsp1/2*, and *nin* mutants, the infection phenotype of the *Mtnf-ya1-1* mutants is less pronounced, as ITs occasionally reach the cortex in this mutant. However, a recent phylogenetic analysis of plant NF-YA proteins revealed that NF-YA1 belongs to a legume-specific subgroup of NF-YA proteins predominantly expressed in roots and nodules and containing a closely related paralogue in *Medicago* called MtNF-YA2 (77% overall amino acid identity and 100% in the conserved DNA- and subunit-binding domains) (Laloum *et al.*, 2013). The existence of a close paralogue of NF-YA1 in *Medicago* raises the possibility of partial functional redundancy between *MtNF-YA1* and *MtNF-YA2* during nodulation. If this is indeed the case, a double *Mtnf-ya1/Mtnf-ya2* mutant could present a much stronger infection phenotype than the single *Mtnf-ya1-1* mutant.

NF-YA1 belongs to a family of DNA-binding proteins that interact in a heterotrimeric TF complex with two histone-like subunits NF-YB and NF-YC. Together these proteins bind CCAAT boxes, as so-called CCAAT box-binding factors (CBFs) (Mantovani, 1999; Petroni *et al.*, 2012; Laloum *et al.*, 2013). Interestingly, in common bean, an NF-YC gene playing a role in both the infection and the organogenetic pathway, with very similar phenotypes to *MtNF-YA1* upon silencing, has recently been described (Zanetti *et al.*, 2010). Identifying NF-YC and NF-YB partners of NF-YA1 involved in infection and nodule organogenesis in *Medicago*, as well as additional proteins interacting with NF-YA1-containing heterotrimeric CBF complexes, would certainly provide new insights into the signalling and developmental pathways controlled by *MtNF-YA1*. The identification of the *Mtnf-ya1-1* null mutant allele will also be instrumental in investigating the molecular targets of MtNF-YA1-containing CBF complexes during rhizobial symbiosis.

## Supplementary data

Supplementary data are available at *JXB* online.

Fig. S1. Expression patterns of *MtNF-YA1* in roots using a p*MtNF-YA1-GUS* reporter construct.

Fig. S2. Expression pattern of *MtNF-YA1* during nodule development using a p*MtNF-YA1-GUS* reporter construct.

Fig. S3. Non-radioactive *in situ MtNF-YA1* mRNA hybridization in 21 dpi nodules: the presence of DIG-labelled RNA probes was assessed using alkaline phosphatase–anti-DIG antibodies.

Fig. S4. Main root-length measurements.

Fig. S5. Morphology and N_2_ fixation capacity of WT and mutant *nf-ya1-1* nodules 53 days post-inoculation.

Fig. S6. Morphology of bacteroids in infected cells observed by transmission electron microscopy.

Fig. S7. Transmission electron microscopy analysis of infection threads (ITs).

Supplementary Data
