# Supplementary material for: The CCAAT box-binding transcription factor NF-YA1 controls rhizobial infection
Source: J Exp Bot. 2013 Dec 6;65(2):481–94. doi: 10.1093/jxb/ert392 (PMC3904707; doi:10.1093/jxb/ert392)
Supplement: Supplementary Data [file supp_65_2_481__index.html]

The CCAAT box-binding transcription factor NF-YA1 controls rhizobial infection — The CCAAT box-binding transcription factor NF-YA1 controls rhizobial infection — Supplementary Data 

# The CCAAT box-binding transcription factor NF-YA1 controls rhizobial infection

## Supplementary Data

Data files

**Files in this Data Supplement:**

- Supplementary Data - Supplementary Data
