# Supplementary material for: The CCAAT box-binding transcription factor NF-YA1 controls rhizobial infection
Source: J Exp Bot. 2013 Dec 6;65(2):481–94. doi: 10.1093/jxb/ert392 (PMC3904707; doi:10.1093/jxb/ert392)
Supplement: Supplementary Data [file supp_ert392_jexbot109405_file001.pdf]

Expression patterns of *MtNF-YA1* in roots using a p-GUS reporter construct

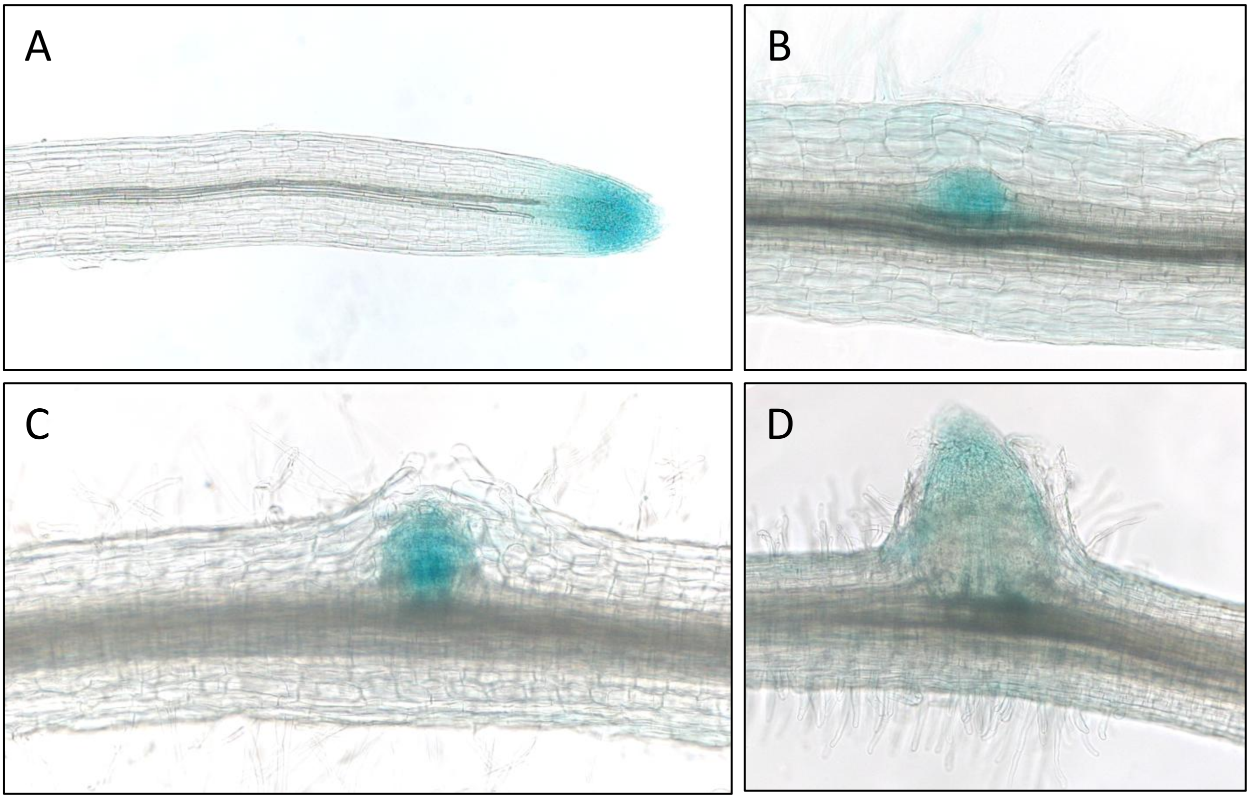

Supplementary Figure S1

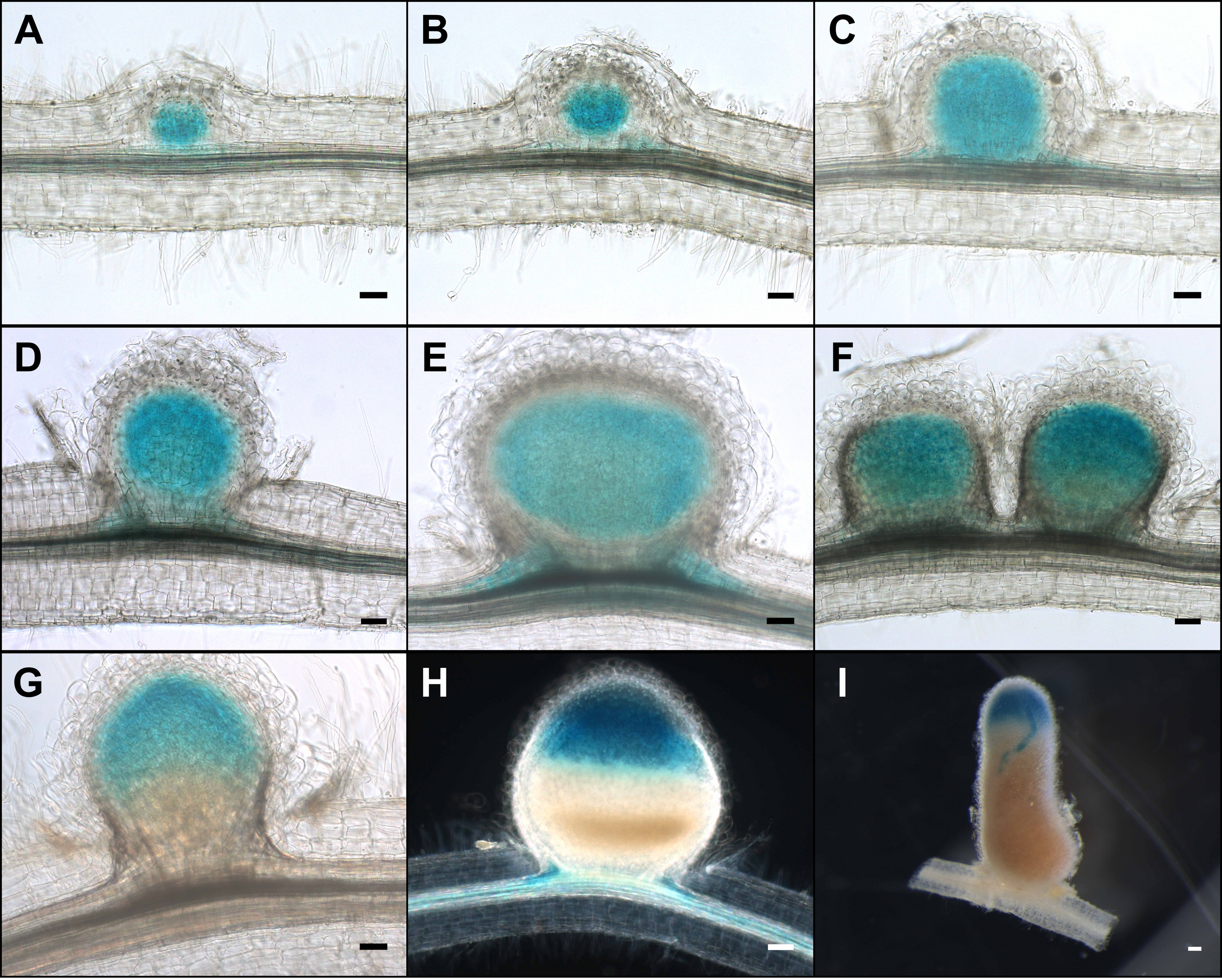

Supplementary Figure S2

non-radioactive *in situ MtNF-YA1* mRNA hybridization in 21dpi nodules

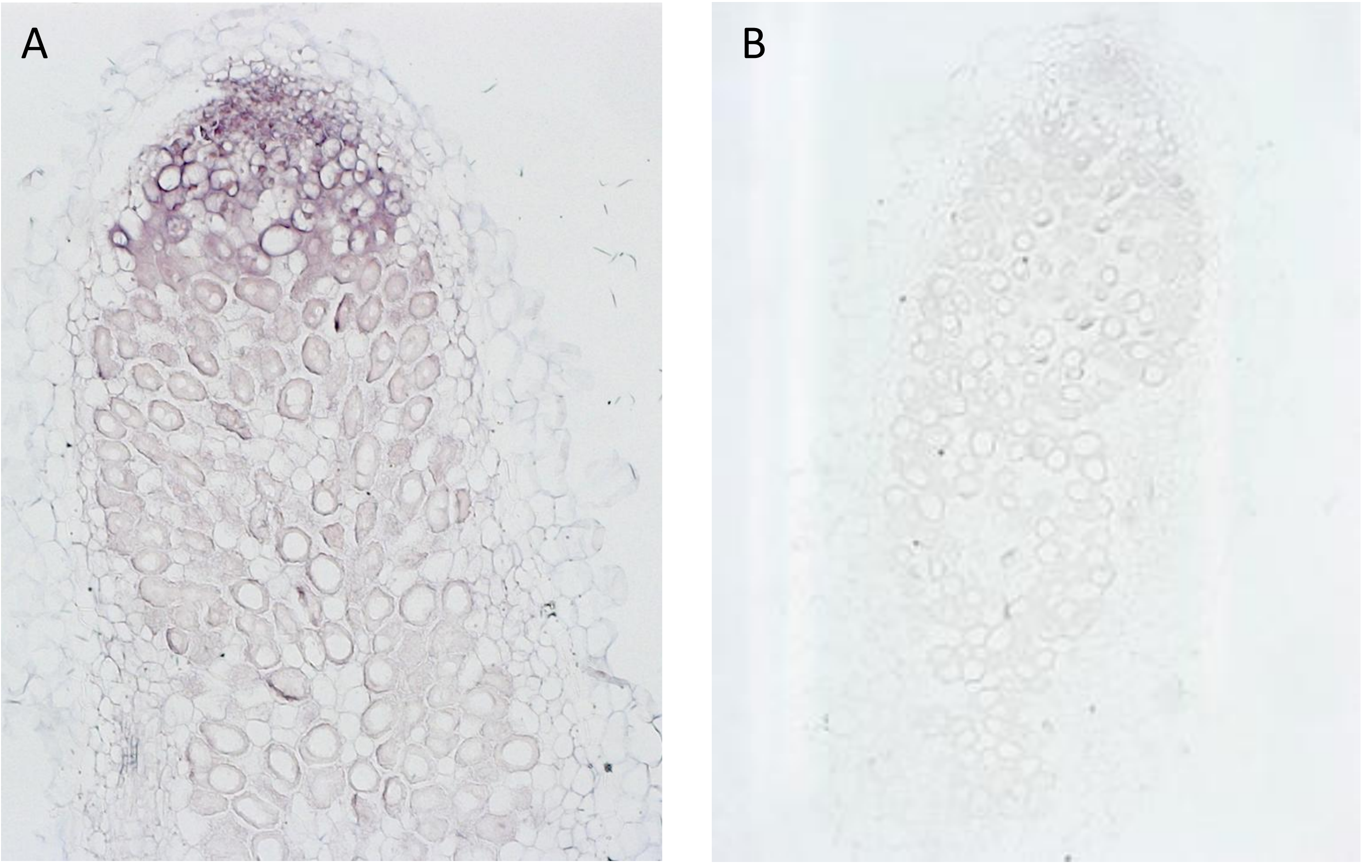

Antisense probe

Sense probe (control)

Supplementary Figure S3

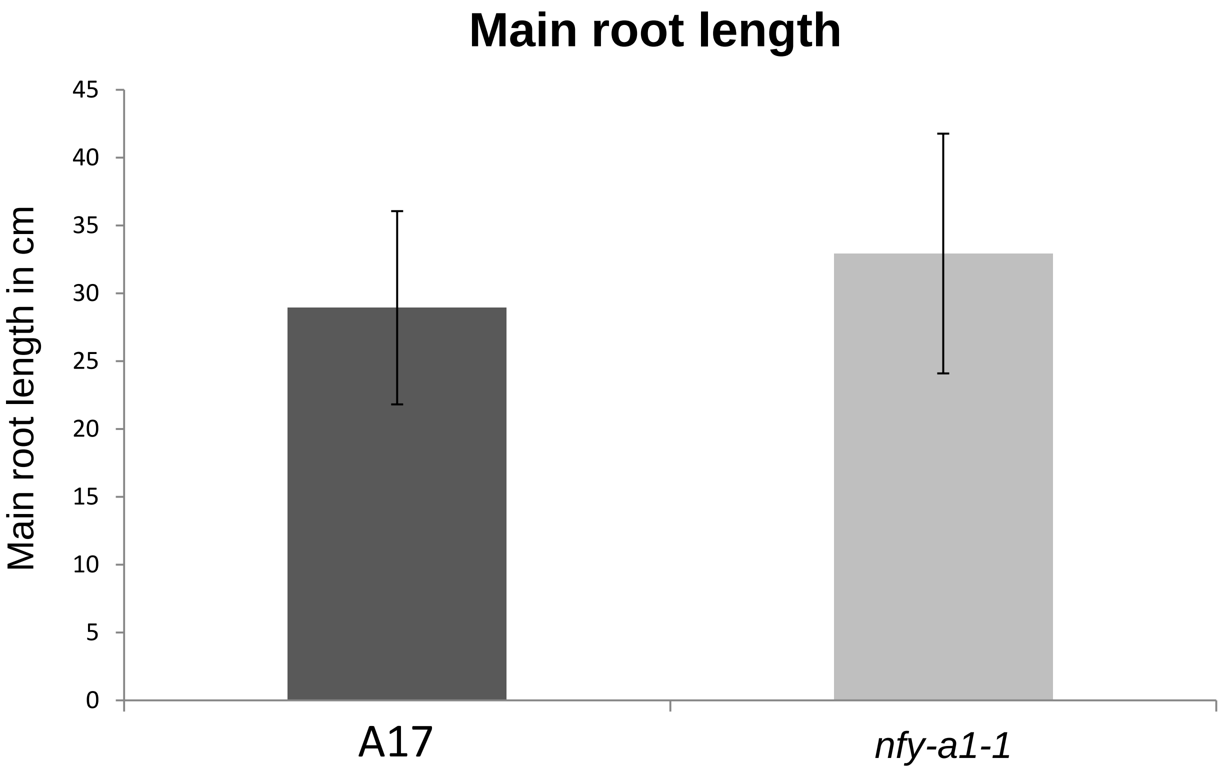

Supplementary Figure S4

Morphology and N2 fixation capacity of WT and mutant *nf-ya1-1 35 dpi* nodules

A

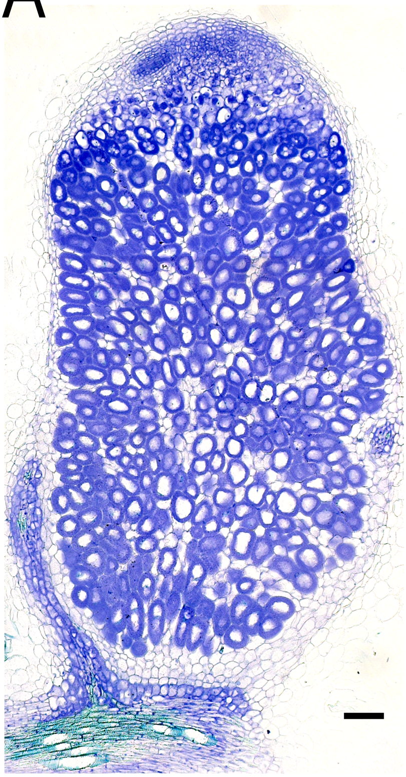

WT A17

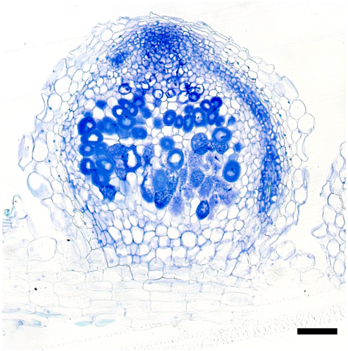

nf-ya1-1

B

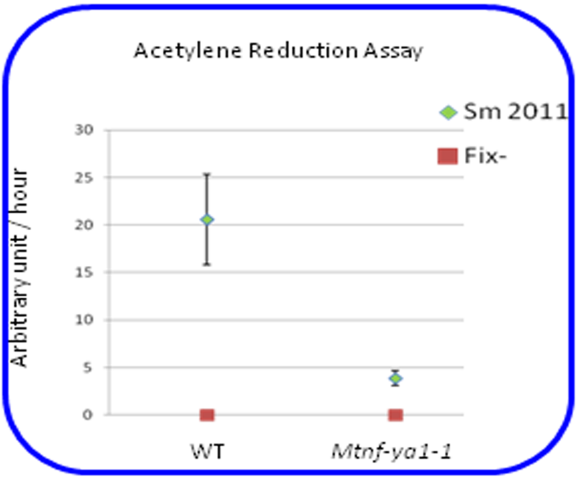

Supplementary Figure S5

Morphology of bacteroids in infected cells

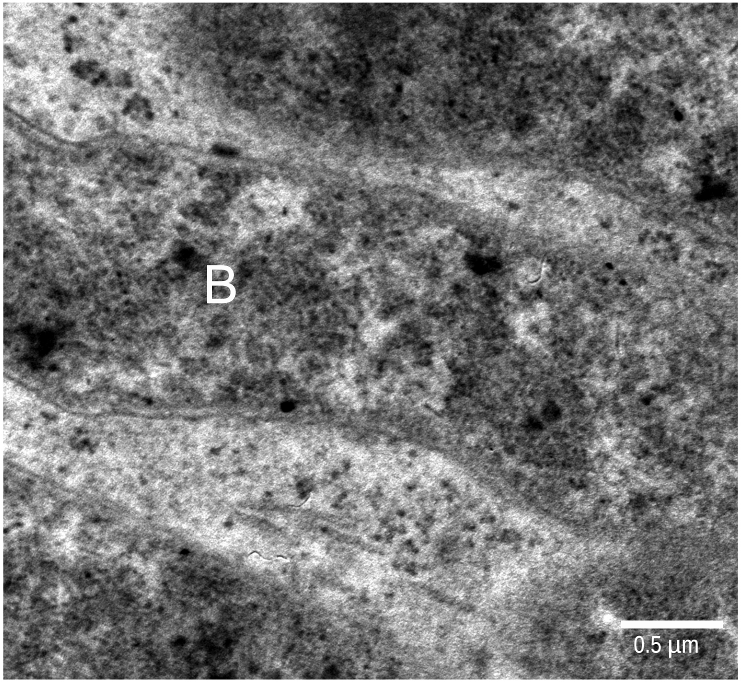

WT A17

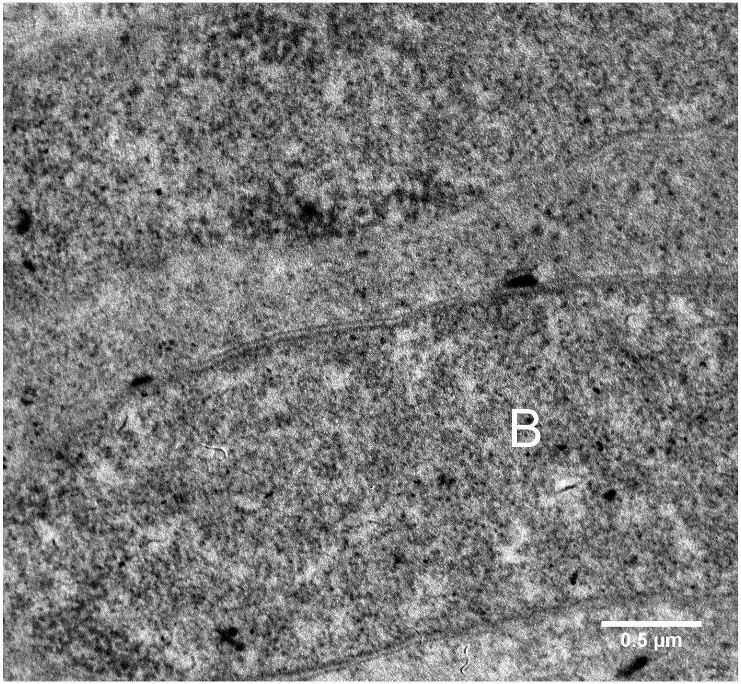

nf-ya1-1 mutant

Supplementary Figure S6

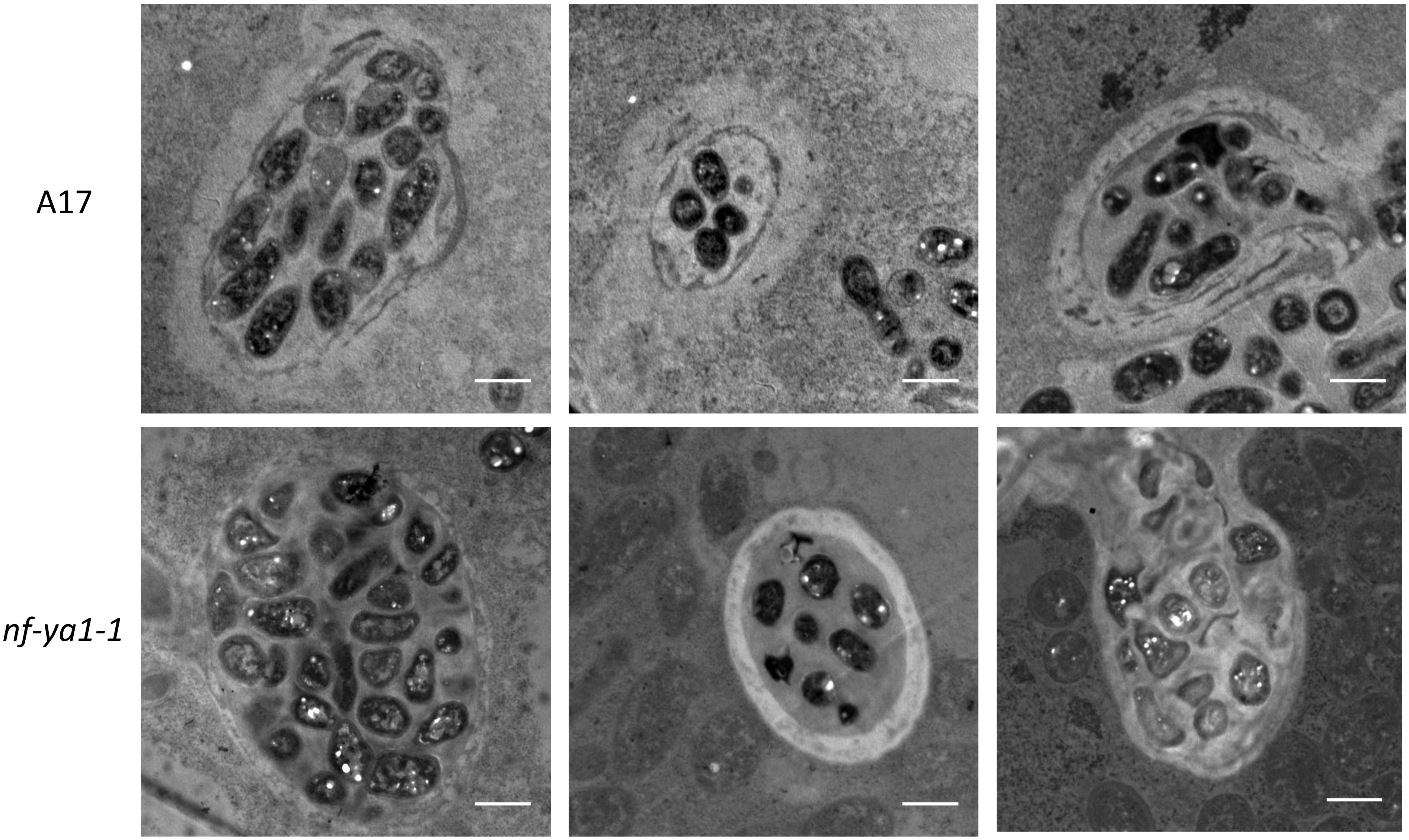

Supplementary Figure 7: Cross section through infection threads(ITs) of the infection zone of 21dpi nodules in WT A17 and in the *Mtnf-ya1-1* mutant. Note the difference in IT wall thickness which is thinner in the mutant compared to WT (Scalebar is 1µm)
